# Application of RSM Method for Optimization of Geraniol Transformation Process in the Presence of Garnet

**DOI:** 10.3390/ijms24032689

**Published:** 2023-01-31

**Authors:** Anna Fajdek-Bieda, Andrzej Perec, Aleksandra Radomska-Zalas

**Affiliations:** Faculty of Technology, Jacob of Paradies University, Chopina 52, 66-400 Gorzow Wielkopolski, Poland

**Keywords:** garnet, RSM, geraniol, selectivity, neral, citronellol

## Abstract

This paper presents the results of tests obtained for the transformation of geraniol in the presence of garnet as a catalyst by the response surface method (RSM). The method analyzed the influence of the following parameters: a temperature of 50–150 °C, a catalyst concentration (garnet) of 1.0–10.0 wt% and a reaction time of 0.25–24 h. Response functions included the conversion of geraniol (GA), selectivity for conversion to neral (NE) and selectivity for conversion to citronellol (CL). In addition, the influence of all control parameters on each of the response parameters is presented in the form of second-order polynomials. The optimal parameters of the geraniol transformation process were a temperature of 55 °C, a catalyst concentration of 5 wt% and a reaction time of 2 h, for which high values of the GA conversion function and the selectivity of conversion to NE and CL were obtained. For the GA conversion, the optimum was obtained at 94 mol% at 60 °C, a catalyst concentration of 5.0 wt% and a reaction time of 2 h. For NE selectivity, the optimum value was reached at 49 mol% at 60 °C, a catalyst concentration equal to 2.5 (5.0) wt% mole and a reaction time of almost 2 h. For CL selectivity, the optimum value of 49 mol% was obtained for control factors: a temperature equal to 20 °C, a catalyst concentration equal to 5.0 wt% and a response time equal to 2 h. The optimal set of control factors for all power factors is characterized by a temperature of 55 °C, a catalyst concentration of 5 wt% and a reaction time of 2 h.

## 1. Introduction

### Garnet and Its Properties

**Figure 1:**
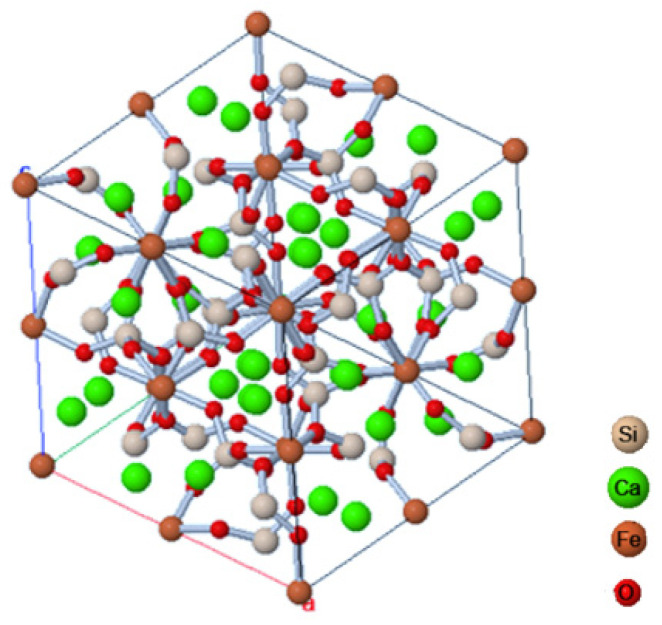
Crystal structure of garnet [2].

Garnets are a group of minerals included in the silicate cluster and are of various elements, mainly magnesium, aluminum, calcium, iron, and manganese. They are divided into several groups based on their chemical composition. Their isometric crystals usually take the form of rhombic icosahedrons and deltoid icosahedrons (Figure 1). They can be transparent, translucent or opaque. They can have different colors such as white, pink, red, yellow, green or black. They are characterized by a vitreous or sometimes burr-like break [1].

Garnet is not a single mineral, but a group of minerals that share the common characteristic of having a crystalline structure and similar chemical composition. The most common varieties available commercially are almandine garnet and andradite garnet. Although almandine garnet is the heaviest and hardest of the garnet group, and andradite garnet is the lightest and softest in the garnet family, both are relatively hard and heavy. In addition to the chemical composition, the process of formation of the mineral also has an impact on the properties of garnet. For example, a large number of crystals in the grain results in greater brittleness, which can be disadvantageous [3]. This tendency results in a higher level of finer particles in the resulting dust and a slightly lower yield at a single use and reduced recyclability. Typically, the lower yield is partially offset by the lower price of andradite garnet compared to almandine.

Asphaltene precipitation in the oil reservoir, production equipment and treatment systems complicate well operations and reduce production. The degree and rate of organic deposition depend on the temperature distribution along the wellbore, the viscosity of the viscous oil, flow velocity, pressure variations and the oil-to-water ratio. Reservoir temperature plays an extremely important role in asphaltene precipitation. In addition, the presence of other organic deposits (such as paraffin and resin) in the oil affects the amount of asphaltene precipitation. Typically, primary asphaltene molecules are considered to have relatively small molecular weights in the range of 500–1000 g/mol with an average of about 750 g/mol, depending on the characteristics of the oil. Analysis of the composition of the asphaltene fraction shows that the amount of carbon and hydrogen usually varies within a small range. However, significant differences occur in the proportions of hetero elements, particularly in the proportions of oxygen and sulfur [4].

The different garnets do not yield the same profile and the same cutting speed and recyclability. Harder, fully crystalline garnets are used in many countries. Almandine garnets achieve faster cutting speeds and lower dust levels than the more brittle, fractured andradite garnets [5,6].

Garnet is also used in the production of bulk products (such as sandpaper and abrasive belts), polishing pastes and aqueous garnet suspensions for precision polishing [7,8].

Almandine garnet is a mineral widely used for sandblasting and is usually derived from sediments (river or beach). There are many advantages to using garnet over other minerals: it can be used for both dry and wet sandblasting [9] and cutting materials [10,11] in a high-pressure water jet (AWJ). Garnets form distinct roughness profiles and virtually do not deposit on the workpiece surface, and for this reason they are used prior to the coating of protective coatings. They are used as an abrasive for cleaning tanks and other confined spaces where a low-dust abrasive is necessary and where water contamination is an issue [12]. They are also widely used to treat aluminum, galvanized and glass surfaces, hangars and in industrial paint shops. These abrasives are also used before powder coating and for demineralizing surfaces [13,14,15].

Garnet is preferred worldwide for its purity of up to 99% in terms of mineralogical content. No chemicals are used during the processing of garnet. The inert nature of this natural mineral ensures that even non-ferrous metal surfaces are not contaminated [16].

Garnet is chemically inert, non-toxic and contains less than 0.2% free silica. It poses no risk of silica or leaching of heavy metal salts such as iron, copper, etc., or radioactive contaminants. Lower consumption and recyclability result in a significantly reduced waste of a non-toxic product [17].

Garnet is an environmentally friendly mineral abrasive. It does not contain any water-soluble components that could contaminate water resources. Used at sea, it does not contaminate the water or seabed, nor does it disturb marine life. It does not pollute the environment due to its inert nature and low dust emissions. It has high recyclability and its lack of toxicity results in better management of its waste. Garnet is a natural mineral that we return to the earth, unlike waste slag abrasives from industrial metal refining, which often contain unacceptable levels of toxic heavy metals such as lead, copper, nickel, zinc, arsenic, cadmium, etc. [18].

As a natural heavy mineral, garnet is effective in softening water. Coarser grains of black garnet are used as filter beds in industrial wastewater treatment and commercial water filtration. Pomegranate is probably the most cost-effective alternative to lodging for water filtration, as the filter bed regenerates faster. In addition, it is also effective in eliminating heavy minerals. It also has the advantage of being chemically inert and being returned to recirculation leads to a longer life in water treatment [19].

In order to optimize the transformation process, response surface methodology (RSM) was applied, which uses methods of mathematical and statistical analysis to determine the interaction between the variables under study allowing the determination of the correct response with a minimum number of experiments.

**Figure 2:**
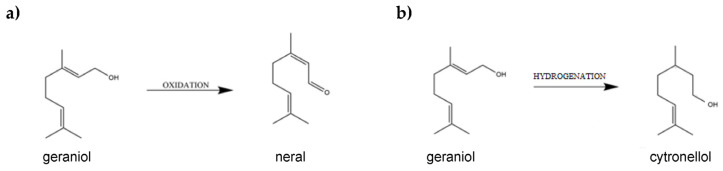
(**a**,**b**) Diagram of the main chemical reactions occurring during the transformation of geraniol in the presence of garnet.

The transformation of geraniol in the presence of garnet produces two compounds: neral (trans-citral, C_10_H_16_O—the product of an oxidation reaction) and citronellol (3,7-Dimethyl-6-Octen-1-Ol, C_10_H_20_O—the product of a hydrogenation reaction) (Figure 2).

Neral (NE) has a distinctive, intense, lemony, sweet fruity odor, perceptible for about 24 h with a sensing threshold of 40 ppb. It is widely used in the cosmetic, pharmaceutical and food industries. In the food industry, it is a flavor and aroma ingredient in the production of alcoholic and non-alcoholic beverages, bakery products, meat products, cheese, ice cream, chewing gum, candy, gelatin and spices in amounts ranging from 1–40 ppm (in the case of chewing gum 200 ppm). Additionally, in the cosmetic and household chemical industry, it is as an aroma (fragrance) in the production of soaps, detergents, creams and lotions and perfumery products. At present, it is produced on an industrial scale by synthetic methods, and only small amounts are obtained by distillation of lemongrass oil or exotic verbena (up to a few tons per year). Among the producers of this compound, both synthetic and natural, we should mention first of all BASF (Germany), Givaudan (Switzerland), Mane (France), Kuraray (Japan), DSM (Netherlands). On the other hand, the technologies for obtaining synthetic citral of the greatest importance include those carried out by BASF and DSM [20,21,22].

Citronellol (CL) is an organic chemical compound characterized by a floral scent. It can be added to cosmetic products to make them fragrant. Depending on the concentration, it can smell of roses or geranium. Citronellol is colorless, insoluble in water, but is well soluble in alcohols. In cosmetics, it is a fragrance substance that is often combined with other compositions. In addition, it masks unpleasant odors, such as sweat. It is a compound found in plants, such as lemongrass, lemon balm and rose. Its origin can also be synthetic. Due to its easy production, it is often an ingredient in many cosmetic products. Citronellol is used in cosmetics to give them fragrance. It is a cheap substance with a pleasant scent, so it is often used in the cosmetic industry. Mainly, it is added to face creams, body lotions, lotions, lotions, bath salts and lozenges, perfumes and eau de toilette, hair shampoos and conditioners, deodorants and antiperspirants, facial cleansers, body massage products, tonics and hydrolats and shaving foams and gels. The use of citronellol in cosmetic products imparts fragrance and also masks unpleasant aromas. It is a popular substance in perfumes, especially those designed for women. Thanks to its sweat odor-masking properties, citronellol is also used in antiperspirants. When various technologies are used to obtain citronellol as a reaction product, either a mixture of (R)-citronellol and (S)-citronellol isomers is obtained (use of traditional hydrogenation catalysts) or both of the aforementioned compounds in the form of pure R and S varieties (use of chiral catalysts, which selectively produce only one of the forms) [23,24].

The response surface methodology (RSM) presented in this paper is innovative for the transformation of geraniol using garnet as a catalyst. To date, the transformation process of geraniol in the presence of pomegranate has not been optimized. The presented method allows the selection of optimal parameters, such as temperature (50–150 °C), amount of catalyst (1–10 wt%) and reaction time (0.25–24 h), considering the high values of conversion of geraniol rates and selectivity of synthesis products (neral and citronellol). The choice of such variation ranges was made on the basis of previous experience, analysis of the state of the issue, as well as the possibility of achieving the technological parameters of conducting research.

## 2. Results and Discussion

A central composite design grounded on RSM was used to design experiments, modeling and process optimization [25]. In addition, for each output parameter, ANOVA was used to assess the significance of the developed models, as well as control parameters. Two values that were analyzed in the ANOVA are the F-value and the corresponding *p*-value. The F-value is the ratio of the mean squares to the mean squares error. The larger the F-value, the greater the variation between sample means relative to the variation within the samples. The *p*-value is the probability of obtaining an F-ratio as large or larger than the one observed, assuming that is no difference between the group averages. 

### 2.1. Impact of Control Factors on GA Conversion

A detailed GA analysis of variance (ANOVA) was performed for a 95% confidence level (α = 0.05) (Table 1 and Table 2). The model factor is significant when it achieves a *p*-Value <0.05 (Figure 2).

Regression equation in uncoded units:

(1) $$CG=30.07+0.8542\;T+6.85\;C+17.18\;\tau -0.0039\;T^{2}-0.0286\;T\cdot C-0.078\;T\cdot \tau -1.287\;C\cdot \tau$$

where:

*CG* is conversion of geraniol [wt%];
*T* is temperature [°C];
*C* is concentration [wt%];
*τ* is time [h].

**Figure 3:**
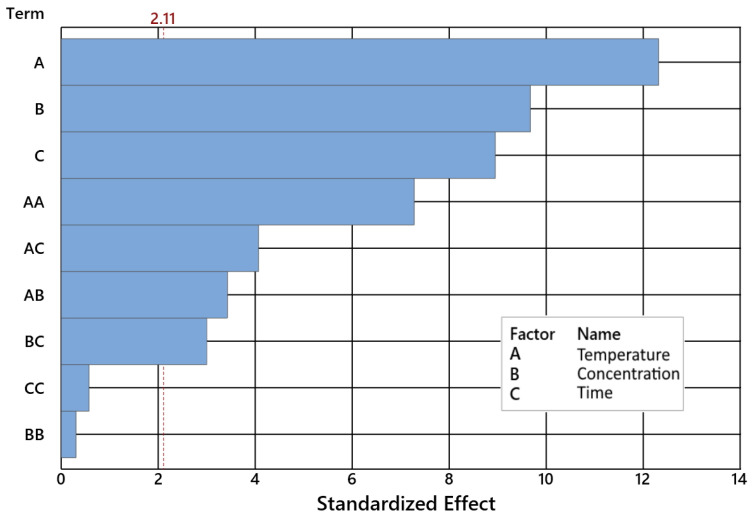
Levels of important and not important factors of the geraniol conversion for α = 0.05.

To approximate the multicollinearity level, the variance inflation factor (VIF) was determined. It quantifies the multicollinearity intensity. No significant multicollinearity was observed for all factors tested, as the VIF belongs to the interval {1, 1.03} (Figure 3).

**Figure 4:**
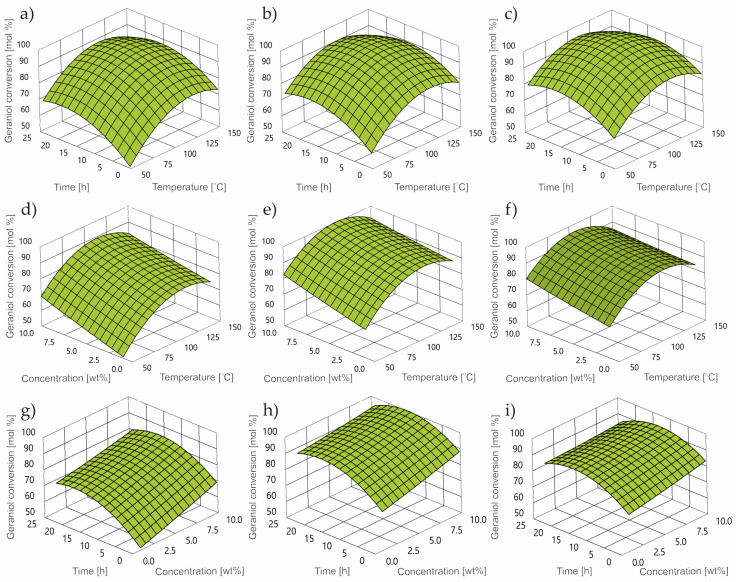
Contour plot of GA conversion at: concentration (**a**) 1 wt%; (**b**) 5 wt%; (**c**) 10 wt%; reaction time (**d**) 0.25 h; (**e**) 3 h; (**f**) 24 h; temperature (**g**) 50 °C, (**h**) 80 °C, (**i**) 150 °C.

The increase of the temperature function to the value of 100 °C causes an increase in the value of geraniol conversion (Figure 4) in the entire range of the tested concentration of the catalyst (garnet). The increase in the concentration of the catalyst causes a proportional increase in the geraniol conversion value in the entire range of the tested temperature and reaction time. In the case of changing the reaction time, the maximum value was reached in the middle of the interval (approx. 12–13 h) for all other parameters.

For all cases, the lowest geraniol conversion values were obtained with the lowest values of control parameters.

### 2.2. Impact of Control Factors on NE Selectivity

**Figure 5:**
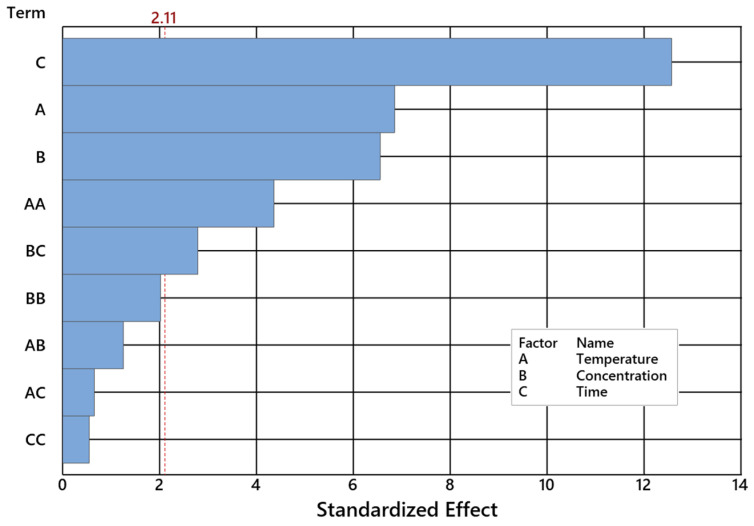
Levels of important and not important factors of the of dimethyl selectivity for α = 0.05.

A detailed NE selectivity analysis was performed by ANOVA for 95% confidence at α = 0.05 (Table 3 and Table 4). Each factor of the model is considerable when its *p*-Value exceeds 0.05 level as presented in Figure 5.

The VIF discloses how the assessed coefficient variance is inflated, as entailed by the multicollinearity occurring in the model. No significant multicollinearity was observed for all factors tested, as the VIF held into the interval {1, 1.03}.

Regression Equation in Uncoded Units

(2) $$DS=-7.41+0.514\;T-1.08\;C+15.55\;\tau -0.003148\;T^{2}-1.603\;C\cdot \tau$$

where:

*DS* is NE selectivity [wt%];
*T* is temperature [°C];
*C* is concentration [wt%];
*τ* is time [h].

**Figure 6:**
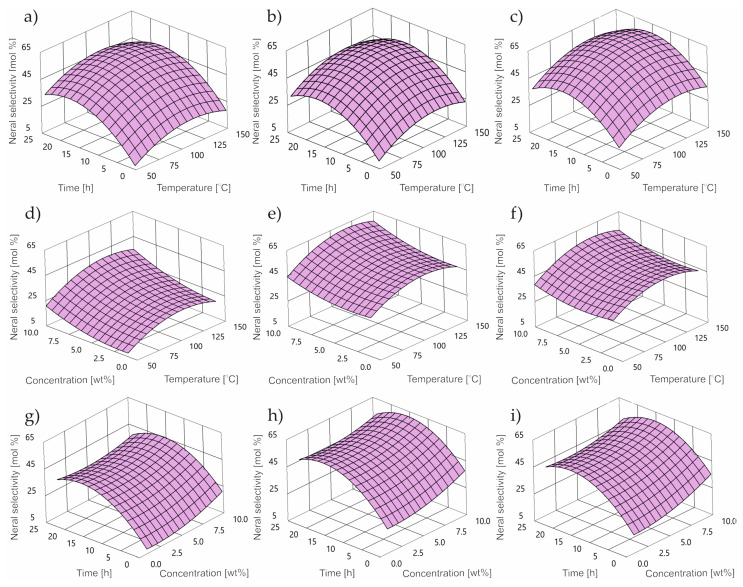
Contour plot of dimethyl selectivity at: concentration (**a**) 1 wt%; (**b**) 5 wt%; (**c**) 10 wt%; reaction time (**d**) 0.25 h; (**e**) 3 h; (**f**) 24 h; temperature (**g**) 50 °C; (**h**) 80 °C; (**i**) 150 °C.

Along with the increase in the value of the temperature function, catalyst concentration and reaction time, an increase in the selectivity of the transformation to neral can be observed (Figure 6) until reaching a maximum in the middle of the range, followed by a decrease.

As the reaction time increases, the neral selectivity values increase rapidly to reach the highest values in the range of 45 to 65 mol%. Minimal values of neral selectivity were observed for low values of all control parameters.

### 2.3. Impact of Control Factors on CL selectivity

**Figure 7:**
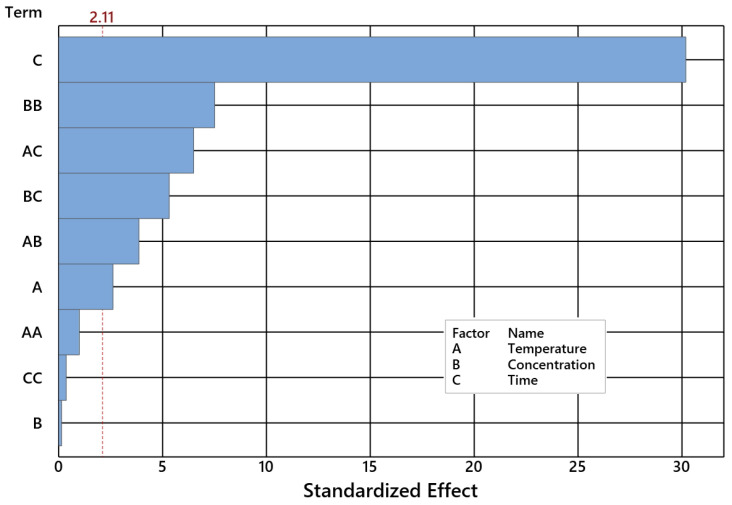
Pareto chart of the standardized effects of CL selectivity plot for α = 0.05.

A detailed CL selectivity analysis was performed by ANOVA for 95% confidence at α = 0.05 (Table 5 and Table 6). Each factor of the model is considerable when its *p*-Value exceeds 0.05 level, as presented in Figure 7. The VIF discloses how the assessed coefficient variance is inflated, as entailed by the multicollinearity occurring in the model. No significant multicollinearity was observed for all factors tested, as the VIF held into the interval {1, 1.03}.

Regression Equation in Uncoded Units

(3) $$TS=13.75+0.0691\;T+18.65\;\tau +1.148\;C^{2}-0.01701\;T\cdot C-0.0659\;T\cdot \tau -1.204\;C\cdot \tau$$

where:

*TS* is thumbergol selectivity wt%;
*T* is temperature °C;
*C* is concentration wt%;
*τ* is time h.

**Figure 8:**
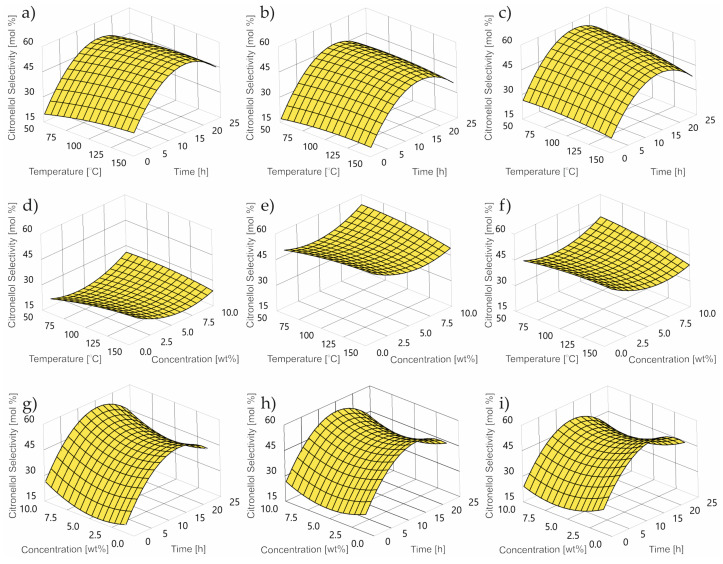
Contour plot of thumbergol selectivity at: concentration (**a**) 1 wt%; (**b**) 5 wt%; (**c**) 10 wt%; reaction time (**d**) 0.25 h; (**e**) 3 h; (**f**) 24 h; temperature (**g**) 50 °C; (**h**) 80 °C; (**i**) 150 °C.

Figure 8a–i demonstrates the impact of the control factors into the value of the CL selectivity level.

In the whole range of the examined control parameters, an upward trend in the value of selectivity of conversion to CL is visible. In terms of response time, this function reaches its maximum at approx. 15 h. In the case of temperature, the selectivity function increases linearly over the entire tested range.

### 2.4. Composite Desirability Coefficient

**Figure 9:**
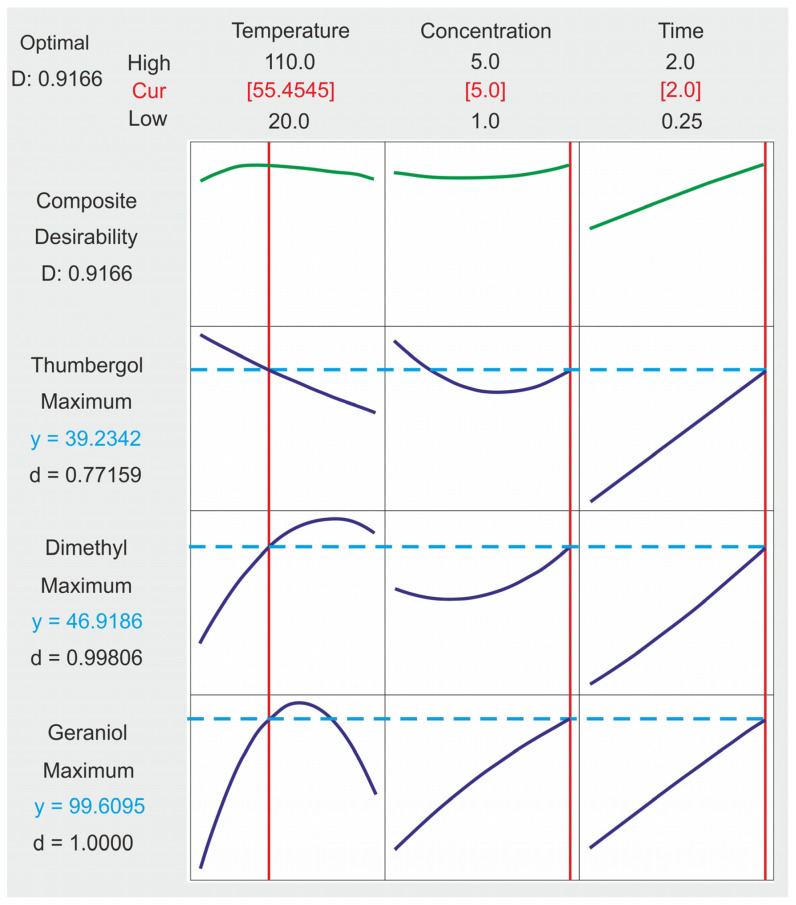
Control factors at optimal levels for each output factor.

Figure 9 presents the results regarding the impact of each of the examined control factors on all output factors. In addition, individual and complex purposefulness was assessed, which shows to what extent the variable meets the set reaction goals. In the model, the purposefulness is close to 1, which indicates that the geraniol transformation process under the suggested conditions achieves appropriate results for each answer. In addition, the optimum for all input parameters was determined: a temperature equal to 55 °C, a catalyst concentration equal to 5 wt% and a reaction time equal to 2 h.

## 3. Materials and Methods

**Figure 10:**
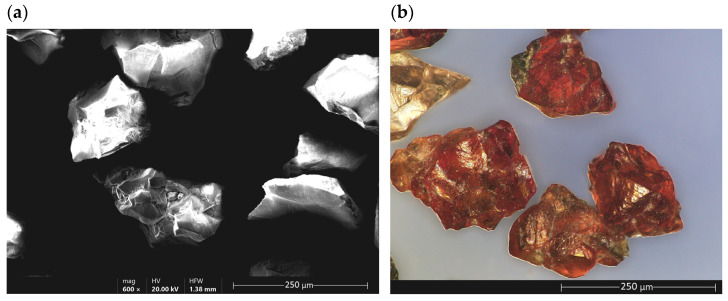
Details of garnet grains: (**a**) SEM view, (**b**) optical microscope view.

Figure 10 shows a microscopic view KEYENCE VHX6000 Digital Microscope, Z100T Lens) of pomegranate grains, whose grains are isometric in shape, with rounded edges and similar dimensions.

**Figure 11:**
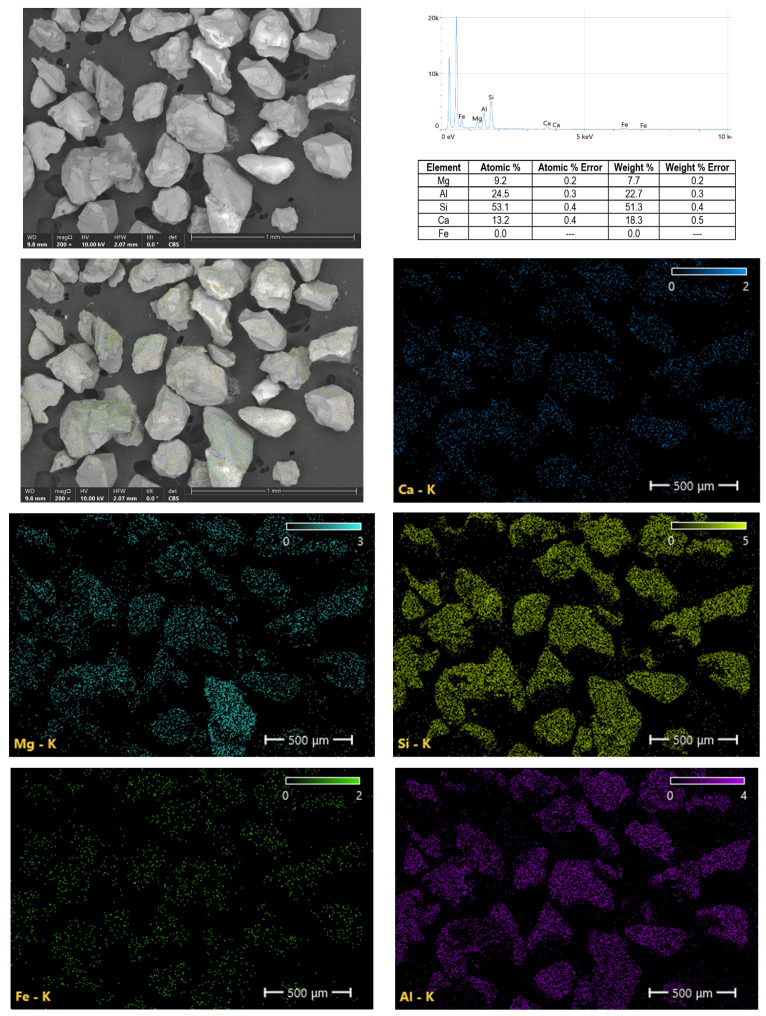
The XRF and EDX spectra of garnet.

Mapping of elements—scanning electron microscopy (SEM) and EDX surface spectraSEM apparatus (Axia ChemiSEM, ThermoFisher, Waltham, MA, USA) with a secondary electron detector. Figure 11 shows maps of the elements contained in the pomegranate sample. Five major elements (magnesium, aluminum, silicon, calcium, iron) were identified in the sample. The element maps made show an even distribution of each of the elements present in the sample.

The following reaction equations were used to evaluate the conversion value of geraniol and the selectivity of conversion to products:

1. GA conversion C_geraniol_:

(4) $$C_{\mathrm{geraniol}}=\frac{\mathrm{amount}\;\mathrm{of}\;\mathrm{moles}\;\mathrm{of}\;\mathrm{geraniol}\;\mathrm{consumed}}{\mathrm{amount}\;\mathrm{of}\;\mathrm{moles}\;\mathrm{of}\;\mathrm{geraniol}\;\mathrm{intrduced}\;\mathrm{into}\;\mathrm{reactor}}\;\cdot 100\%$$

2. Selectivity to the key products (NE and CL) S_product/geraniol_:

(5) $$S_{\mathrm{product}/\mathrm{geraniol}}=\frac{\mathrm{amount}\;\mathrm{of}\;\mathrm{moles}\;\mathrm{of}\;\mathrm{product}}{\mathrm{amount}\;\mathrm{of}\;\mathrm{moles}\;\mathrm{of}\;\mathrm{geraniol}\;\mathrm{consumed}}\;\cdot 100\%$$

The control parameters and their ranges are as follows: temperature (50–150 °C), amount of catalyst (1–10 wt%) and reaction time (0.25–24 h).

In order to shorten the number of experiments performed, the design of experiment (DOE) methodology was used [26]. The experiments were conducted using a factorial design—response surface methodology (RSM), which consists of 27 tests (Table 7). Each of the tests conducted was repeated three times. The individual variables and the examined response of the process analysis were performed by ANOVA for 95% confidence at α = 0.05. The RSM method is a statistical and mathematical method for modeling combinations, which considers the relationships between the relevant variables and the studied process response [27,28,29].

Statistica software was used to develop the model equations. The effects of the independent variables on the GA conversion function and the selectivity of NE and CL (dependent variables) are shown in Table 7.

Columns (2–4) show the values of the control parameters (inputs) for the research process, while columns (5–7) present the values of the results (output parameters).

The second-degree equation for determining the regression model value is:

(6) $$y=\beta_{0}+\overset{k}{\underset{i=1}{\sum }}\beta_{i}x_{i}+\overset{k}{\underset{i=1}{\sum }}\beta_{ii}x_{i}^{2}\pm \varepsilon$$

where:

*y* is the dependent variable (response);
*x_i_* shows values of the *i*-th cutting parameter;
*β*_0_, *β_i_*, *β_ii_* are the factors of regressions;
*ε* is the error acquiring in the cutting.

## 4. Conclusions

The use of the surface response method (RSM) in the process of geraniol transformation in the presence of garnet as a process catalyst made it possible to determine which of the tested process parameters actually affect the course of the reaction, while allowing the omission of those factors that have a marginal impact on the effects. Optimization of the control factors (temperature, catalyst concentration and reaction time) made it possible to determine their values to obtain the maximum values of the tested functions (GA conversion, NE and CL selectivity) and to determine the interactions between the factors of the tested functions. The conducted optimization studies allowed the following conclusions to be drawn:

- For the GA conversion, the optimum was obtained at 94 mol% at 60 °C, a catalyst concentration of 5.0 wt% and a reaction time of 2 h.
- For NE selectivity, the optimum value was reached at 49 mol% at 60 °C, a catalyst concentration equal to 2.5 (5.0) wt% mole and a reaction time of almost 2 h.
- For CL selectivity, the optimum value of 49 mol% was obtained for control factors: a temperature equal to 20 °C, a catalyst concentration equal to 5.0 wt% and a response time equal to 2 h.
- The optimal set of control factors for all power factors is characterized by a temperature of 55 °C, a catalyst concentration of 5 wt% and a reaction time of 2 h.

It turned out that the application of the RSM method allowed for the minimization of research procedures, shortening the time needed to obtain appropriate results, and reducing the cost of research by reducing the necessary number of tests.

## Figures and Tables

**Table 1:** Model summary of GA conversion.

S	R^2^	R^2^(adj)	R^2^(pred)
1.96809	97.64%	96.39%	93.37%

**Table 2:** ANOVA results for geraniol conversion.

Source	DF	Adj SS	Adj MS	F-Value	*p*-Value
Model	9	2722.82	302.535	78.11	0.000
Linear	3	1662.14	554.047	143.04	0.000
Temperature [°C]	1	776.45	776.449	200.46	0.000
Catalyst concentration [wt%]	1	432.38	432.376	111.63	0.000
Time [h]	1	517.47	517.470	133.60	0.000
Square	3	665.65	221.882	57.28	0.000
Temperature [°C]*Temperature [°C]	1	605.61	605.615	156.35	0.000
Catalyst concentr. [wt%]*Catalyst concentr. [wt%]	1	0.71	0.714	0.18	0.673
Time [h]*Time [h]	1	59.32	59.317	15.31	0.001
Two-Way Interaction	3	165.49	55.162	14.24	0.000
Temperature [°C]*Catalyst concentration [wt%]	1	45.44	45.439	11.73	0.003
Temperature [°C]*Time [h]	1	69.46	69.465	17.93	0.001
Catalyst concentration [wt%]*Time [h]	1	50.58	50.582	13.06	0.002
Error	17	65.85	3.873		
Total	26	2788.67			

**Table 3:** Model summary of NE selectivity.

S	R^2^	R^2^(adj)	R^2^(pred)
3.74216	93.89%	90.65%	83.95%

**Table 4:** Analysis of variance of NE selectivity.

Source	DF	Adj SS	Adj MS	F-Value	*p*-Value
Model	9	3657.94	406.44	29.02	0.000
Linear	3	2825.31	941.77	67.25	0.000
Temperature [°C]	1	518.48	518.48	37.02	0.000
Catalyst concentration [wt%]	1	419.92	419.92	29.99	0.000
Time [h]	1	1986.79	1986.79	141.88	0.000
Square	3	618.42	206.14	14.72	0.000
Temperature [°C]*Temperature [°C]	1	358.51	358.51	25.60	0.000
Catalyst concentr. [wt%]*Catalyst concentr. [wt%]	1	82.51	82.51	5.89	0.027
Time [h]*Time [h]	1	177.40	177.40	12.67	0.002
Two-Way Interaction	3	95.49	31.83	2.27	0.117
Temperature [°C]*Catalyst concentration [wt%]	1	11.97	11.97	0.85	0.368
Temperature [°C]*Time [h]	1	3.61	3.61	0.26	0.618
Catalyst concentration [wt%]*Time [h]	1	79.91	79.91	5.71	0.029
Error	17	238.06	14.00		
Total	26	3896.00			

**Table 5:** Model summary of CL selectivity.

S	R^2^	R^2^(adj)	R^2^(pred)
2.09472	96.54%	94.71%	91.22%

**Table 6:** Analysis of variance of CL selectivity.

Source	DF	Adj SS	Adj MS	F-Value	*p*-Value
Model	9	2083.41	231.49	52.76	0.000
Linear	3	1616.44	538.81	122.80	0.000
Temperature [°C]	1	2.48	2.48	0.57	0.462
Catalyst concentration [wt%]	1	0.00	0.00	0.00	0.990
Time [h]	1	1616.21	1616.21	368.34	0.000
Square	3	465.68	155.23	35.38	0.000
Temperature [°C]*Temperature [°C]	1	7.75	7.75	1.77	0.201
Catalyst concentr. [wt%]*Catalyst concentr. [wt%]	1	199.48	199.48	45.46	0.000
Time [h]*Time [h]	1	258.45	258.45	58.90	0.000
Two-Way Interaction	3	136.52	45.51	10.37	0.000
Temperature [°C]*Catalyst concentration [wt%]	1	106.70	106.70	24.32	0.000
Temperature [°C]*Time [h]	1	26.64	26.64	6.07	0.025
Catalyst concentration [wt%]*Time [h]	1	3.18	3.18	0.72	0.406
Error	17	74.59	4.39		
Total	26	2158.00			

**Table 7:** Details of the conducted tests.

Test nr.	Temp	Catalysts Concentration	Time	GA Conversion	NE Selectivity	CL Selectivity
*-*	[°C]	[wt%]	[h]	[mol%]	[mol%]	[mol%]
1	20	1.0	0.25	40	7	13
2	20	1.0	1.00	51	20	31
3	20	1.0	2.00	73	38	41
4	20	2.5	0.25	60	12	13
5	20	2.5	1.00	68	19	29
6	20	2.5	2.00	80	28	37
7	20	5.0	0.25	73	19	27
8	20	5.0	1.00	78	26	36
9	20	5.0	2.00	85	39	49
10	60	1.0	0.25	73	14	23
11	60	1.0	1.00	80	29	33
12	60	1.0	2.00	87	41	46
13	60	2.5	0.25	79	20	20
14	60	2.5	1.00	84	28	30
15	60	2.5	2.00	85	49	40
16	60	5.0	0.25	86	37	27
17	60	5.0	1.00	91	43	33
18	60	5.0	2.00	94	49	40
19	110	1.0	0.25	78	22	31
20	110	1.0	1.00	79	28	32
21	110	1.0	2.00	88	40	45
22	110	2.5	0.25	80	19	19
23	110	2.5	1.00	85	27	29
24	110	2.5	2.00	86	48	39
25	110	5.0	0.25	87	36	26
26	110	5.0	1.00	91	42	32
27	110	5.0	2.00	92	48	39

## Data Availability

Not applicable.
